# CYP1B1 knockout enhanced IFN-γ production is required but not sufficient for protection of cigarette smoke-exposed mice against lethal influenza virus infection

**DOI:** 10.3389/fimmu.2025.1600025

**Published:** 2025-07-04

**Authors:** Wenxin Wu, Jeremy S. Alexander, J. Leland Booth, Magdalena Chlebicz, Craig A. Miller, Chao Xu, Susan Kovats, Jordan P. Metcalf

**Affiliations:** ^1^ Pulmonary, Critical Care & Sleep Medicine, Department of Medicine, University of Oklahoma Health Sciences Center, Oklahoma City, OK, United States; ^2^ Arthritis & Clinical Immunology Program, Oklahoma Medical Research Foundation, Oklahoma City, OK, United States; ^3^ Department of Veterinary Pathobiology, College of Veterinary Medicine, Oklahoma State University, Oklahoma State University, Stillwater, OK, United States; ^4^ Department of Biostatistics and Epidemiology, University of Oklahoma Health Sciences Center, Oklahoma City, OK, United States; ^5^ Department of Microbiology and Immunology, University of Oklahoma Health Sciences Center, Oklahoma City, OK, United States; ^6^ Veterans Affairs Medical Center, Oklahoma City, OK, United States

**Keywords:** influenza virus, CYP1B1, innate, smoking, IFN-γ, inflammation

## Abstract

**Background:**

Cigarette smoke (CS) exposure increases the frequency and severity of respiratory influenza A virus (IAV) infections in humans and increases mortality in mice. There is evidence that Cytochrome P450 family 1 subfamily B member 1 (CYP1B1) enhances lung injury during hyperoxic exposure in animal models. We used an *in-vivo* mouse model to assess the hypothesis that CYP1B1 modifies innate immune responses to CS and alters survival during IAV infection.

**Methods:**

We measured CYP1B1 induction in bronchial epithelial cells in smokers and nonsmokers obtained by bronchoscopy by whole transcriptome analysis. To determine whether CYP1B1 knockout (CYP KO) improves mortality and reduces lung injury in CS-exposed mice, we compared the survival rates, host immune responses, and lung-to-body weight ratio of CYP KO mice with the wild-type (WT) mice following challenge with IAV with or without CS exposure.

**Results:**

CYP1B1 is one of the most highly upregulated genes in human lung epithelia derived from cigarette smokers. In CS-exposed mice, CYP1B1 knockout (CYP KO) significantly increased survival during IAV infection. In both nonsmoking (NS) and CS mice, CYP KO significantly enhanced IAV-induced increases in total immune cell numbers in bronchoalveolar lavage fluid (BALF) without causing additional lung injury. CYP KO caused a more rapid IFN-γ response to IAV infection in lungs from both NS- and CS-exposed mice. Specifically, we found that IFN-γ was significantly increased in BALF and in the lung at day 5 post-infection (p.i.) in these mice. Flow cytometry showed that innate lymphocytes produced early IFN-γ in the lungs of KO mice. We confirmed the importance of IFN-γ for CYP KO survival by adding IFN-γ antibody to IAV-infected CYP KO mice. IFN-γ antibody treatment of CS-exposed CYP KO mice completely abolished the improved survival rate seen in KO mice without IFN-γ antibody treatment. However, IFN-γ administration to CS-exposed WT mice did not increase survival rates as seen in CS-exposed CYP KO mice after IAV infection.

**Conclusions:**

Our results demonstrate that CYP KO in mice protects against CS-enhanced susceptibility of smokers during influenza infection. IFN-γ is required but not sufficient for the protection of CS-exposed CYP KO mice against lethal IAV infection.

## Introduction

Influenza A virus (IAV) infection is a major cause of infectious morbidity and mortality (1). IAV is a highly contagious respiratory pathogen that triggers a robust innate immune response upon infection. Once the virus enters the respiratory tract, it is recognized by pattern recognition receptors (PRRs) such as Toll-like receptors (TLRs) and RIG-I-like receptors (RLRs), which detect viral RNA (2, 3). This recognition rapidly activates signaling pathways that lead to the production of pro-inflammatory cytokines and interferons (IFNs). Key cytokines such as IFN-β, IFN-γ, IL-6, TNF-α, and IL-1β play critical roles in orchestrating the early immune response, recruiting immune cells like natural killer (NK) cells, neutrophils, and macrophages to the site of infection (4–6). The balance and timing of cytokine responses are crucial in determining the severity of IAV infection (7–9).

Cigarette smoking is a significant public health problem (10). It is the primary cause of chronic obstructive pulmonary disease (COPD) and predisposes those with COPD to severe respiratory tract infections (11). Cigarette smoke (CS) exposure alone increases the frequency and severity of respiratory tract infections (12), increases the risk of influenza hospitalization, and reduces influenza vaccine effectiveness in the elderly (13). There is evidence that innate and adaptive responses to influenza infection are altered in CS patients, which was confirmed in CS animal models (14). Studies using animal models suggest that CS may contribute to severe infection outcomes by impairing the antiviral response during the initial stages of infection. Additionally, CS exposure increases mortality and prolongs recovery in IAV infection (15–18).

As a major site for environmental exposure to inhaled toxicants, the respiratory tract contains many enzymes that metabolize these compounds. A major class of proteins expressed in the bronchial and alveolar epithelia are the cytochrome p450 (CYP) enzymes, of which there are 50 isoforms (20, 21). Although CYP enzymes ideally metabolize xenobiotics to decrease toxicity, this is not always the case. For example, exposure to dioxin stimulates CYP enzymes that convert arachidonic acid to hydroxyeicosatrienoic acids (HETEs), which play an important role in inflammation (22). CYP1A1 and CYP1B1 also convert inhaled polycyclic aromatic hydrocarbons to carcinogens (23). There is also evidence that CYP enzymes, specifically CYP1B1, enhance lung injury during hyperoxic exposure in animal models. CYP1B1 knockout (CYP KO) mice had ~50% less lung injury (lung weight/body weight) and lung inflammation than wild-type (WT) mice after 48h–72h hyperoxic exposure (24). In the human bronchial epithelial BEAS-2B cell line, CYP1B1 expression also played a role in hyperoxia induced-cytotoxicity. Although it has not been studied in detail, alterations of cytokine expression may play a role in decreased barrier function induced by CYP1B1. Cytokine IL-6 and VEGF mRNA induction by hyperoxia is inhibited in CYP KO mice, and VEGF contributes to lung injury caused by other stimuli (25). With regard to viral infections, CYP1A1 is suppressed by Coxsackie B infection in mice, CYP3A4 is suppressed in hepatocytes infected with adenovirus, while other CYP enzyme subtypes are variably induced or suppressed by hepatitis viruses (reviewed in (26)). Though CYP enzymes are known to be affected by IAV infection in mice and vaccination in humans (27, 28), to our knowledge there is a notable lack of studies investigating the role of CYP1B1 in IAV infection across both human and mouse models.

A previous study showed that treatment of cells with e-cigarette aerosol induced CYP1B1 mRNA and protein in a human oral keratinocyte cell line (29). Genetic polymorphisms in CYP1B1 have been linked to altered lung cancer risk in smokers, with certain variants modifying the relationship between tobacco carcinogen exposure and cancer susceptibility (30). These findings highlight CYP1B1 as a potential biomarker and therapeutic target in cigarette smoke-related lung diseases. In this report, we assessed CYP1B1 expression in bronchial epithelial cells from smokers and nonsmokers using whole transcriptome analysis of samples obtained via bronchoscopy. We then evaluated the *in vivo* whole animal relevance of the results in humans by determining if global CYP1B1 deficiency affects outcomes during IAV infection, particularly with CS exposure, and the mechanisms involved. Outcomes assessed included survival rates, host immune responses, and lung injury.

## Materials and methods

### Ethics statement

The Institutional Animal Care and Use Committee (IACUC) of the University of Oklahoma Health Sciences Center approved all of the protocols for the animal experiments (protocol number: 17-106-HI). A total of 244 mice were used for this manuscript. The facility where this research was conducted is accredited by AAALAC. The facility operates according to the Guide for the Care and Use of Laboratory Animals and the requirements of the Animal Welfare Act and Regulations, and the Public Health Service Policy on Humane Care and Use of Laboratory Animals. All procedures were performed by personnel trained in the techniques according to IACUC guidelines. All invasive clinical procedures were performed while animals were anesthetized. In compliance with the Animal Welfare Act principles, we reduced the number of animals sacrificed by omitting control groups for which comparative effects are already known. The animals were sacrificed by an overdose of isoflurane with a secondary method of euthanasia. This study followed the recommendations in the ARRIVE (Animal Research: Reporting of *In Vivo* Experiments) guidelines.

### Isolation of primary human bronchial epithelial cells

Human bronchial epithelial cells (HBEC) were obtained by bronchoscopy and bronchial brushing with written, informed consent from both smoking and non-smoking, healthy, adult volunteers in accordance with a protocol approved by the institutional review board of the University of Oklahoma Health Sciences Center (IRB # 2197). The smokers had a smoking history of at least 10 pack years with ½ to 1 pack of cigarettes per day. All subjects were between the ages of 25 and 60. The 20 subjects consisted of 10 smokers, 5 males and 5 females, and 10 nonsmokers, 5 males and 5 females. All smoking and nonsmoking participants were matched by gender, age, and ethnicity. Three or four separate bronchi were brushed, and the cells were rinsed from the brush into 10-ml sterile saline until 5 × 10^6^ to 1 × 10^7^ cells total were collected as determined by hemocytometer counts for total and viable cells using trypan blue exclusion. The HBECs were centrifuged at 400 × g for 5 min. Cells were resuspended to 5 × 10^5^ cells/ml in complete Bronchial Epithelial Cell Growth Medium (BEGM; Lonza Group Ltd.), were seeded into collagen-coated tissue culture plates (Bio-Coat, BD Biosciences) at a density of 1 × 10^5^ cells/cm^2^, and were propagated in an incubator at 37°C in 5% CO_2_. After 24h, the cells were washed with HBSS to remove non-adherent cells, and fresh complete BEGM was added. When the cultures were near confluence (7–10 days), cells were harvested for RNA extraction.

### Influenza a virus and mouse infection

The IAV strain used in this study was A/PR/34/8 (PR8). The stocks were propagated in Madin-Darby canine kidney (MDCK, ATCC, and Manassas, VA) cells following standard procedures (31). The virus was titered by plaque assay in MDCK cells, aliquots were made, and stored at −80°C. LD_50_ for each viral preparation was determined as described (8).

We obtained C57BL/6 CYP1B1 knockout mice (gift of Dr. F. Gonzalez) from NIH (32). Littermates of CYP1B1 knockout and WT mice of both sexes were analyzed at 12–14 weeks of age. Mice were held in a vertical position while sedated and infected by intranasal instillation of PR8 virus diluted in PBS (50 µl solution). An equal volume of PBS without virus instilled intranasally was used as a control in the mock group. All infected animals were sacrificed by an overdose of isoflurane at 5 days post-infection (p.i.). The animals were meticulously watched both during and after each procedure to make sure they recovered properly. Mice were monitored daily for up to 16 days for clinical symptoms (shivering, inactivity, hunched posture, and piloerection), and their weight was recorded daily, or until the experimental endpoint, whichever came first.

### Whole-body CS exposure

Mice were exposed to the smoke of 1R6F reference cigarettes (University of Kentucky, Lexington, KY) for 4h per day (19). Mice receiving CS were gradually brought up to the target exposure over a period of 2 weeks and treated 5 days/week for 6 weeks. Treatment was administered by placing mice in a Plexiglas smoking chamber (Teague Enterprises, Davis, CA). Smoke exposure was standardized to total suspended particles = 90 mg/m^3^, 11% mainstream and 89% sidestream smoke in the chamber of the machine. The exposure level was assessed by measuring serum cotinine, a nicotine metabolite, at 1h after exposure (Cotinine ELISA Kit, GenWay Biotech Inc.). After six weeks of exposure, the average serum cotinine was 513 ± 256 ng/ml, near levels in human cigarette smokers (33). “Nonsmoking” (NS) treatment groups were conducted for the same periods of time, but mice were exposed to filtered room air.

### Bronchoalveolar lavage

Mice were sacrificed using isoflurane. BAL was performed using a closed thorax technique by exposing the trachea, nicking the bottom of the larynx, and inserting a 3/4-inch 22-gauge cannula into the proximal trachea. The proximal end of the trachea was tied off, and 0.6 ml of sterile PBS was gently introduced into the lungs and recovered. This was repeated 3 times for a total instilled volume of 1.8 ml. Return volume varied by < 10% between samples. The total cell count in BAL fluid (BALF) was determined using a hemocytometer. BALF was then centrifuged to remove cells and was stored at −20°C. Total protein in BALF was determined by a Pierce BCA Protein Assay Kit (Thermo Fisher Scientific, Waltham, MA).

### Multiplex immunoassay

Cytokine protein levels in the bronchoalveolar lavage fluids (BALF) were determined by multiplex immunoassay (Eve Technologies, Calgary, AB, Canada). All samples were twofold diluted in 1% Triton X-100 (final) for inactivation of residual IAV.

### Measurement of mRNA expression by quantitative real-time polymerase chain reaction

Total RNA from the lung was extracted using a modified TRIzol (Invitrogen, Carlsbad, CA) protocol and spectrophotometrically quantified. The integrity of RNA was verified by formaldehyde agarose gel electrophoresis. Equal amounts (1 µg) of RNA from each sample were reverse-transcribed into cDNA with oligo (dT) SuperScript II First-Strand Synthesis System for real-time polymerase chain reaction (RT-PCR) (Invitrogen, Carlsbad, CA). Gene-specific primers for mouse PRRs, cytokines, and the β-actin housekeeping genes were used. The primers’ sequences were described in our earlier publication (34). qRT-PCR was performed using 100 ng sample RNA and SYBR Green (Quanta Biosciences, Gaithersburg, MD) in a Bio-Rad CFX96™ Touch Real-Time PCR Detection System. Results were calculated and graphed from the ΔCt of the target gene and normalizer, β-actin.

### Microarray analysis

Initial HBEC RNA quality control was performed for each sample with Affymetrix Expression Console 1.4.1.46. All RNA samples were found to have RNA Integrity Numbers greater than 4.5, electropherograms indicated high-quality RNA, and all 20 samples were retained. The samples were amplified using the GeneChip^®^ WT PLUS Reagent Kit (Thermo Fisher Scientific). The concentration of single‐stranded cDNA produced by amplification was measured using a NanoDrop 2000 (Thermo Fisher Scientific). RNA was amplified into ss‐cDNA, fragmented, labeled, and hybridized (Thermo Fisher Scientific GeneChip^®^ Hybridization, Wash, and Stain Kit) onto GeneChip Human Transcriptome Assay 2.0 microarrays (Thermo Fisher Scientific) for analysis. Microarrays were hybridized for 18h, rotating at 60 rpm at 45°C. Chips were then washed and stained using two GeneChip^®^ fluidics station 450 (Affymetrix), using protocol FS450‐0001, as per the HTA 2.0 microarray protocol. Stained and washed microarrays were scanned using a 7G‐modified GeneChip^®^ Scanner 3000 (Affymetrix). Differential expression detection was performed using R/limma in R for analysis and subgroups of interest.

### Histological and immunohistochemistry analysis of mouse lung

Mice were euthanized at day 5 after IAV infection, and lungs were fixed in 4% paraformaldehyde in PBS for 24 hours at room temperature before being embedded in paraffin. Paraffin-embedded sections were trimmed to 5 μm according to previously published methods and guidelines (35, 36) and collected onto charged slides before staining with hematoxylin and eosin (H&E) for microscopic evaluation by light microscope. Lung tissues were evaluated for alveolar damage, hyaline membrane formation, serous exudate/edema, alveolar fibrin deposition, alveolar histiocytes, perivascular infiltrates, type II pneumocyte hyperplasia, peribronchial inflammation, smooth muscle hyperplasia, thrombosis, and fibrinoid vasculitis. All tissues were assigned a quantitative histopathological score based on previously described criteria (37, 38): 0 = no apparent pathology/change, 1 = minimal change (minimally increased numbers of inflammatory cells), 2 = mild change (mild inflammatory infiltrates, damage/necrosis, fibrin deposition, and/or exudation), 3 = moderate change (more moderately extensive than 2), and 4 = marked changes (severe inflammation, damage/necrosis, exudation, vasculitis, and/or thrombosis). All tissues were evaluated and scored by a board-certified veterinary pathologist (CAM) blinded to study groups to eliminate bias and ensure scientific rigor.

### Isolation of cells from lung tissue

Lungs were perfused with PBS + 1 mM EDTA before digestion for 60 min with Liberase TM (0.1 mg/ml) and DNase I (0.1 mg/ml) (all from Roche) in PBS + 0.5% BSA pH 7.4 (17). Lung cells were filtered (70 μM), washed with RPMI + 10% FCS or HBSS without Ca^2+^ and Mg^2+^, respectively, and red cells lysed using RBC lysis buffer (BD Biosciences).

### Flow cytometry

For surface staining, cells were incubated with monoclonal antibodies (mAbs) on ice for 15 min after 5 min of anti-CD16/32 treatment in FACS buffer (PBS, 5% newborn calf serum, 0.1% NaN3). Myeloid cells in the lung were identified with fluorochrome-labeled mAbs: CD45.2-FITC (104), IFN-γ-PE (XMG1.2), CD11c-PECy7 (N418), Ly6G-BV786 (1A8), CD11b-PercpCy5.5 (M1/70), MHCII-AF700 (M5/114), SiglecF-BV421 (E50-2440), Ly6C-PEDazzle594 (HK1.4), CD24-BV605 (M1/69), and CD64-APC (X54-5/7.1). Lymphocytes in the lung were identified with fluorochrome-labeled mAbs: CD3-PECy7 (145-2C11), CD4-PercpCy5.5 (GK1.5), CD8-APC (53-6.7), NK1.1-APC/Fire750 (PK136), and CD19-PEDazzle594 (6D5). An intracellular cytokine staining kit (BD Biosciences) was used according to the manufacturer’s instructions with IFN-γ-PE (XMG1.2). mAbs were purchased from BD Biosciences, BioLegend, Tonbo Biosciences, and Thermo Fisher Scientific. Live/dead cell discrimination was done with a fixable Zombie Aqua™ dye (BioLegend, San Diego, CA). Samples were acquired on an LSRII instrument containing four lasers (BD Biosciences) and analyzed using FlowJo software (Treestar, Ashland, OR).

### Statistical analysis

Statistical significance was determined by one-way ANOVA with a Tukey test for multiple comparisons. Survival rate significance was determined by the log-rank test. Statistical analysis between two groups was also performed by an unpaired two-tailed student t-test. For RT-PCR results, the *p*-value was calculated from the ΔCt values from different experimental groups. Significance was considered as *p* < 0.05.

## Results

### CYP1B1 is highly expressed in HBECs from cigarette smokers and cigarette smoke extract–treated lung epithelial cells

**Figure 1:**
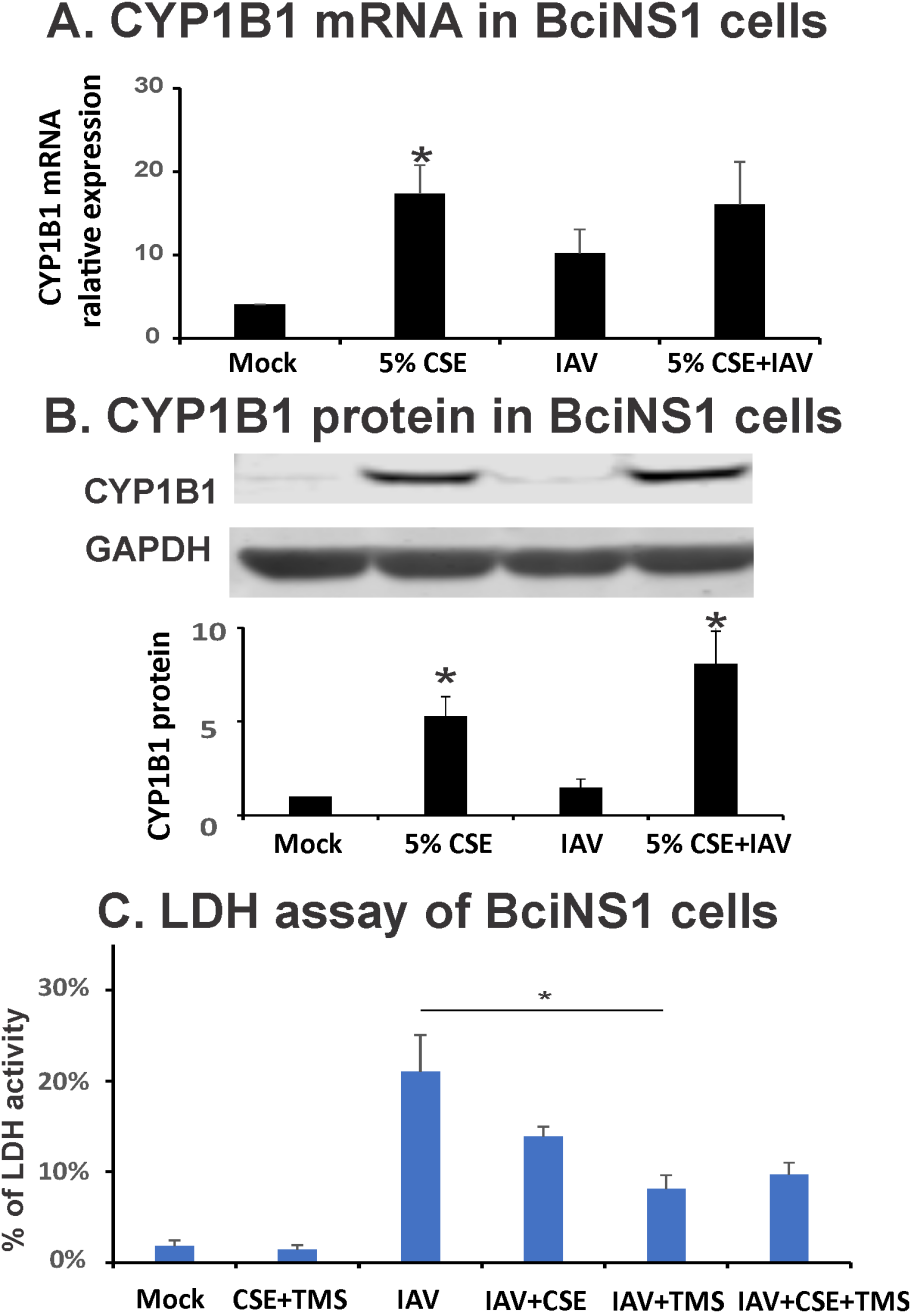
CYP1B1 is induced by IAV and CSE in BciNS1 cells, and TMS protects airway epithelial cells from cell death induced by IAV infection. BciNS1 cells were incubated with 0% (Mock) and 5% CSE for 24h. For IAV infection, the cells were exposed to IAV PR8 at an MOI of 0.1 for 24h. **(A)** CYP1B1 mRNA levels were assessed by qRT-PCR and normalized to β-actin. **(B)** CYP1B1 protein expression in BciNS1 cells was determined by western blot. Data are expressed as means ± SD. * denotes a significant difference compared to the mock group (*P* < 0.05 by one-way ANOVA with Tukey test for multiple comparisons, *n* = 3.). **(C)** LDH activity in the supernatant and cell extract was measured using an LDH Cytotoxicity Assay Kit (BioVision Research Products). Cytotoxicity is expressed as the percentage of supernatants released LDH to total (supernatant + cell) LDH. * denotes a significant difference between the two groups (*P* < 0.05 by one-way ANOVA with Tukey test for multiple comparisons, *n* = 3).

As CS exposure worsens outcomes during IAV infection in mice and humans, we asked whether CS exposure modulated expression of any genes that could predispose to lung injury during IAV infection. We isolated HBECs from 10 normal nonsmokers and 10 age (± 5 years), sex, and ethnicity-matched smokers we recruited through an IRB-approved protocol. RNA was isolated from freshly isolated cells, and whole transcriptomic analysis by microarrays was performed, and the results were analyzed (**Table 1**). Of the ten genes most upregulated in smokers, we noted that CYP1B1 is known to play a role in hyperoxia-induced lung injury and inflammation (24). This gene is induced sixfold (FDR 0.0001) in smokers versus nonsmokers and has the second greatest induction of any gene in these subjects (**Table 1**). We further confirmed the induction of CYP1B1 in BciNS1 cells, which are human airway epithelial cells derived from human airway basal cells via expression of a retrovirus expressing human telomerase (39). We exposed BciNS1 cells to 5% cigarette smoke extract (CSE), IAV (MOI = 0.1), or both. CSE exposure induced CYP1B1 gene expression 4.1-fold, and IAV infection caused a 2.5-fold induction (**Figure 1A**). CSE treatment or IAV infection led to a significant increase in CYP1B1 protein levels within these human airway epithelial cells (**Figure 1B**). IAV infection can cause airway epithelial cells to die through apoptosis, necrosis, and pyroptosis. We assessed cell viability by measuring the percentage of lactate dehydrogenase (LDH) release. Remarkably, tetramethylsilane (TMS), a CYP1B1 inhibitor, protects airway epithelial cells from cell death induced by IAV infection in both CSE-treated and nontreated cells (**Figure 1C**). CYP1B1 is also upregulated by CS or incense smoke in mice and rats, as shown by other groups (40, 41). Thus, our data and other groups’ results led us to investigate whether CYP1B1 plays a role in lung injury in CS-exposed mice during IAV infection.

**Table 1:** Ten most upregulated genes in human bronchial epithelial cells (HBECs) isolated from smokers.

Gene symbol	Gene name	Fold-change	FDR (adj *P*-value)
AKR1B10	aldo-keto reductase family 1, member B10 (aldose reductase)	8.53	1.190E-04
CYP1B1	cytochrome P450, family 1, subfamily B, polypeptide 1	5.98	4.393E-03
SLC7A11	solute carrier family 7 (anionic amino acid transporter light chain, xc- system), member 11	4.79	1.639E-03
CEACAM5	carcinoembryonic antigen-related cell adhesion molecule 5	3.62	2.012E-03
AKR1C2	aldo-keto reductase family 1, member C2	3.50	5.533 E-05
CYP4F11	cytochrome P450, family 4, subfamily F, polypeptide 11	3.44	6.118E-04
CABYR	calcium binding tyrosine-(Y)-phosphorylation regulated	3.30	1.739E-04
ADH7	alcohol dehydrogenase 7 (class IV), mu or sigma polypeptide	3.26	1.045E-07
MUCL1	mucin-like 1	3.06	1.144E-04
ALDH3A1	aldehyde dehydrogenase 3 family, member A1	3.01	3.982E-07

HBECs were isolated from 10 normal nonsmokers and 10 age (± 5 years), sex, and ethnicity-matched smokers recruited through an IRB-approved protocol. RNA was isolated from freshly isolated cells, and whole transcriptomic analysis was performed, and the results were analyzed. Fold-change is from smokers (*n* = 10) versus nonsmokers (*n* = 10). Of the 10 most upregulated genes, CYP1B1 is induced sixfold (FDR 0.0001) in smokers versus nonsmokers, and has the second greatest induction of any gene in these subjects.

### CYP KO improves survival in CS-exposed mice during lethal IAV infection

**Figure 2:**
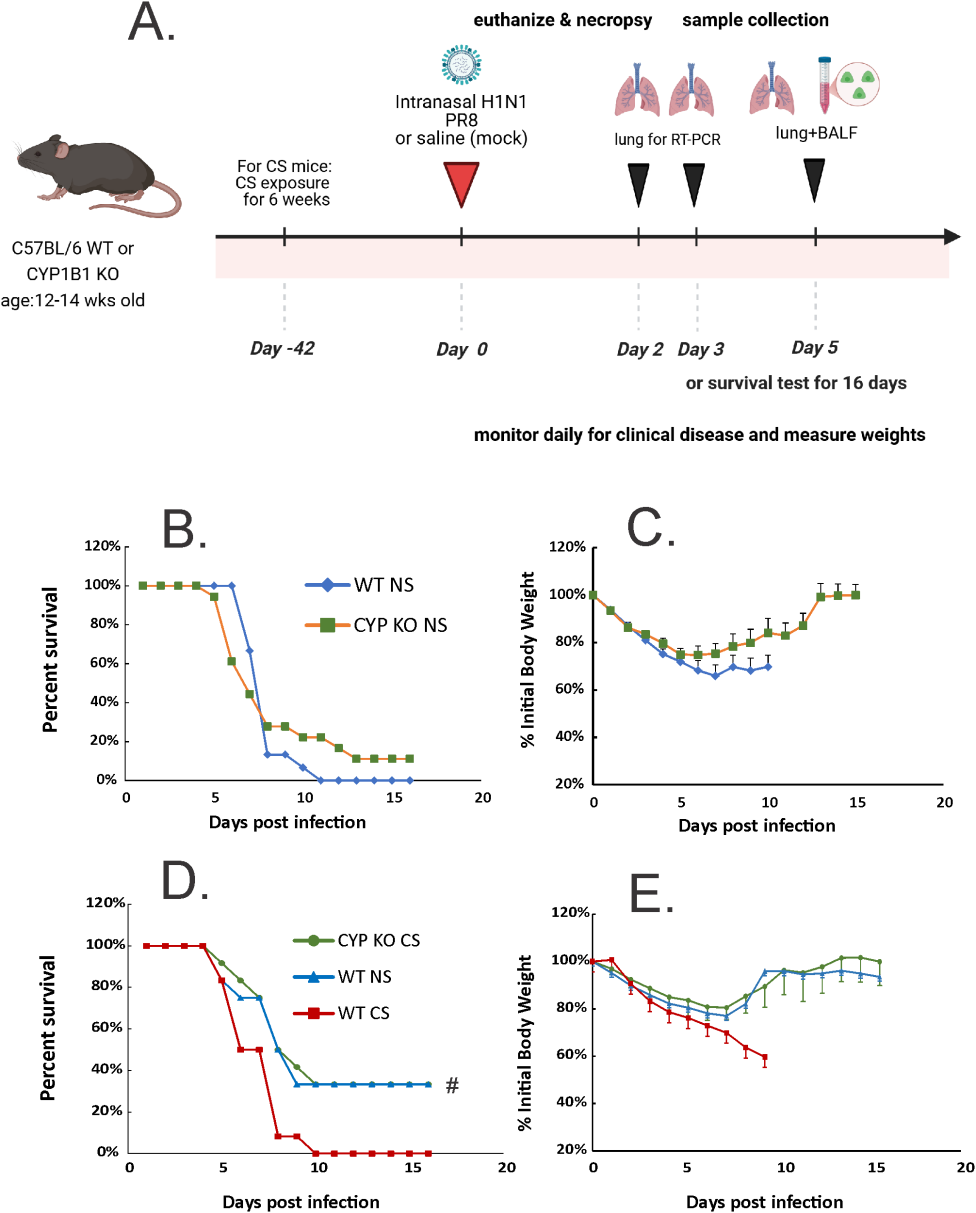
CYP KO increased survival rates of both NS and CS-exposed mice in lethal IAV infection. **(A)** Schematic of the experimental plan on CS exposure and IAV infection. The mice were intranasally inoculated with IAV at 2000 **(B, C)** or 1000 **(D, E)** PFU/mouse. Mortality and body weight were monitored daily. Body weight data were normalized to each mouse’s starting body weight. Data are expressed as mean ± standard deviation (*n* ≥ 12 for each group; total mice = 70 in this experiment). Mortality **(B)** and body weight **(C)** during lethal IAV infection in NS mice. Mortality **(D)** and body weight **(E)** during lethal IAV infection in CS mice. # denotes significant survival rate difference between the CYP KO CS and WT CS groups, *p* < 0.05. Survival rate significance was determined by log rank test.

First, to test whether CYP1B1 knockout (CYP KO) improves mortality in NS mice, we inoculated animals with a lethal dose of PR8 IAV (2000 PFU; **Figure 2A**). Death was recorded when mice were found dead in the cage or at 70% of their original body weight. CYP KO mice tended to have decreased mortality, although the survival curve difference did not reach statistical significance (**Figure 2B**). Weight loss occurred in both infected groups before the first mouse died on day 5 (**Figure 2C**). Again, the body weight reduction of CYP KO mice appeared to be less than that of WT mice, although the difference did not reach statistical significance.

We next sought to investigate CYP KO effects in CS-exposed mice. Whole-body CS exposure was performed as described (19). Briefly, mice were exposed to CS for 4h per day for 6 weeks in a smoking chamber. We determined the LD_5.0_ of IAV PR8 in WT mice (1000 PFU/mouse), and all mice were inoculated with this dose (**Figures 2D, E**). As we have previously shown (8, 34) CS exposure increased morbidity and mortality of IAV infection in mice, with CS-exposed mice having a much lower survival rate than NS mice after IAV infection (**Figure 2C**; 0% for WT CS vs. 40% for WT NS mice). However, CYP KO mice exposed to CS had significantly improved survival compared to CS-exposed WT mice during IAV infection (40% survival for CYP KO CS vs. 0% for WT CS, *p* < 0.05, log-rank test). The survival rate of CS-exposed CYP KO mice improved to that seen in WT NS mice. Morbidity was also decreased, as CYP KO mice had likely less weight loss compared to WT mice at days 8 and 9 post-infection (**Figure 2E**), with weight loss being restored back to that seen in WT NS IAV-infected mice. Thus, the data demonstrated that CYP KO significantly alleviated mortality in CS-exposed mice during IAV infection.

### CYP KO increased immune cell influx in BALF and less lung injury in NS mice during IAV infection

**Figure 3:**
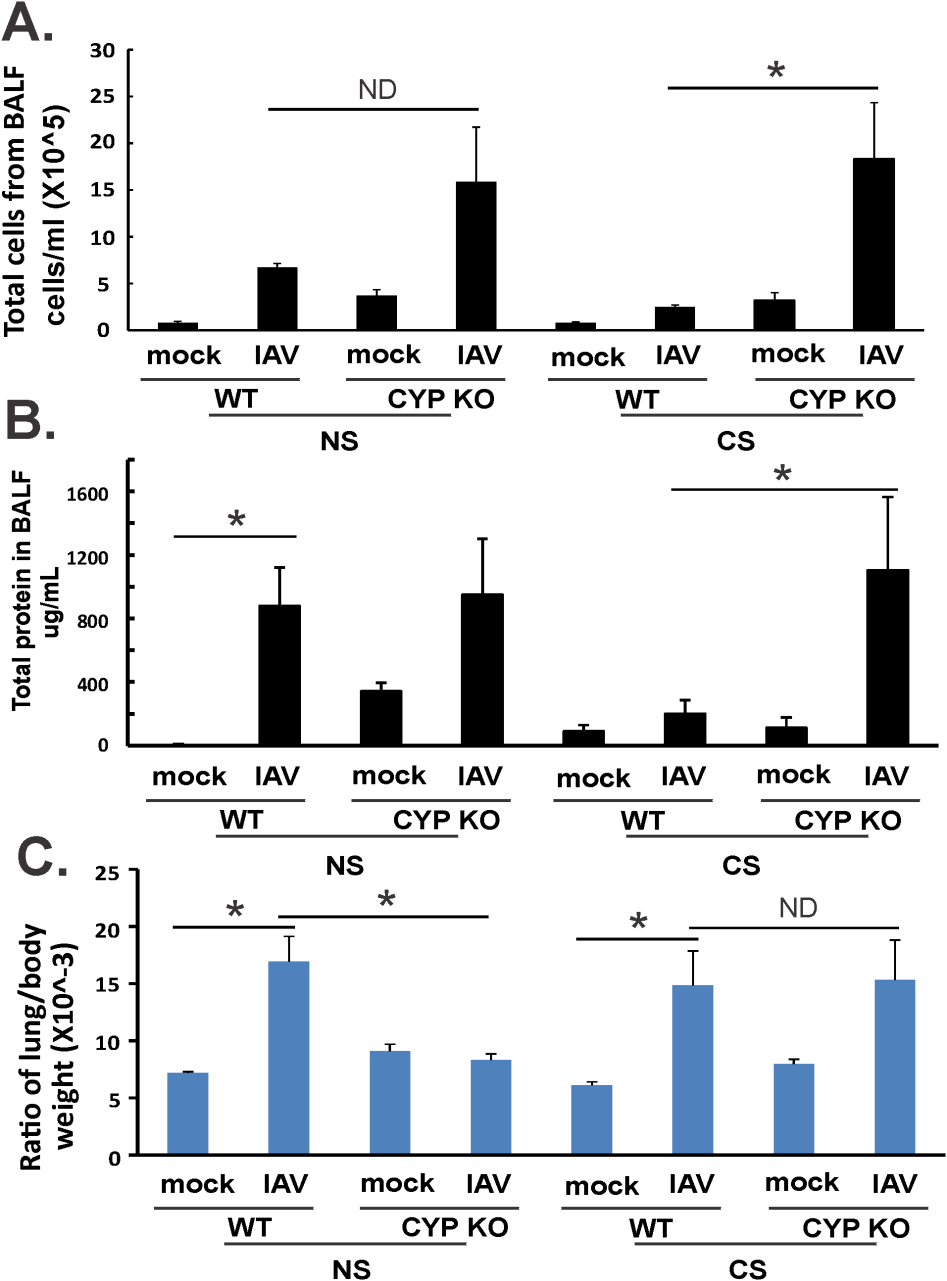
Lung injury and BALF cellularity. Each mouse was infected intranasally with 500 PFU of IAV. Mock-treated mice were inoculated with PBS. Bronchoalveolar lavage fluid (BALF) or lung tissue was harvested at day 5 after infection. Total immune cells **(A)**, total protein concentration **(C)** in BALF, and ratio of lung/body weight **(B)** were determined. Data are expressed as means ± SEM (*n* = 5/group; total mice = 40 in this experiment). ^*^denotes significant difference between the two groups, *p* < 0.05. ND, no significant difference between the two groups. Statistical significance was determined by one-way ANOVA with the Tukey test for multiple comparisons. NS, nonsmoking.

We then asked whether CYP KO decreased lung injury during IAV infection. Mice were inoculated intranasally with IAV at 500 PFU/mouse. The mock group was sham inoculated with an equal volume of PBS as a negative control. Animals were euthanized by overdose of isoflurane at 5 days p.i. We first determined the total inflammatory cell numbers in bronchoalveolar lavage fluids (BALF). IAV infection increased the total viable leukocyte number in BALF in WT NS mice. This increase was further enhanced in CYP KO mice infected with IAV even after they were exposed to CS. The increase in BALF leukocyte number happened in CS-exposed CYP KO mice (**Figure 3A**) even though CS decreased total BALF cell numbers during IAV infection in WT mice in these experiments, consistent with our prior studies (34). These results showed that CYP KO increased immune cell influx into the lung in IAV-infected mice. The total amount of protein in BALF serves as an indicator of lung inflammation as well as permeability, with elevated levels signifying increased leakage of proteins from the blood vessels into the lung alveoli, often due to conditions like lung inflammation or acute lung injury. The total amount of protein in the BALF was increased in CS CYP KO groups relative to similarly treated CS WT mice after IAV infection (**Figure 3B**). These data indicated that an impairment of recruitment mediated by CS was likely related to higher mortality of CS-exposed IAV-infected WT mice. However, CYP KO mice recruited more immune cells to the lung, and the presence of higher immune cells could potentially correct the innate immune response impairment caused by CS. Notably, an intriguing observation is the increased levels of inflammatory cells and protein content in CYP KO mice at baseline within the NS group, indicating that CYP KO may predispose the mice to an immune alert state (**Figures 3A, B**).

The lung-to-body weight ratio (LBR) is relevant in assessing lung health and function, serving as an indicator for lung injury due to pulmonary edema, as a significantly elevated LBR compared to normal values suggests an excessive amount of fluid in the lungs, which is the hallmark of pulmonary edema (42, 43). In WT mice, IAV infection significantly increased LBR in both NS and CS mice (**Figure 3C**). However, CYP KO significantly decreased this ratio to that seen in mock-infected WT mice. In CS mice, LBR in CYP KO and WT mice were similar after infection (**Figure 3C**). Thus, in NS mice, CYP KO increased cellular influx and decreased lung injury compared to levels seen in IAV-infected WT mice. In CS-exposed mice, KO increased cellular influx and did not cause more lung injury than WT mice.

### Effect of CYP KO on lung histopathology during IAV infection

**Figure 4:**
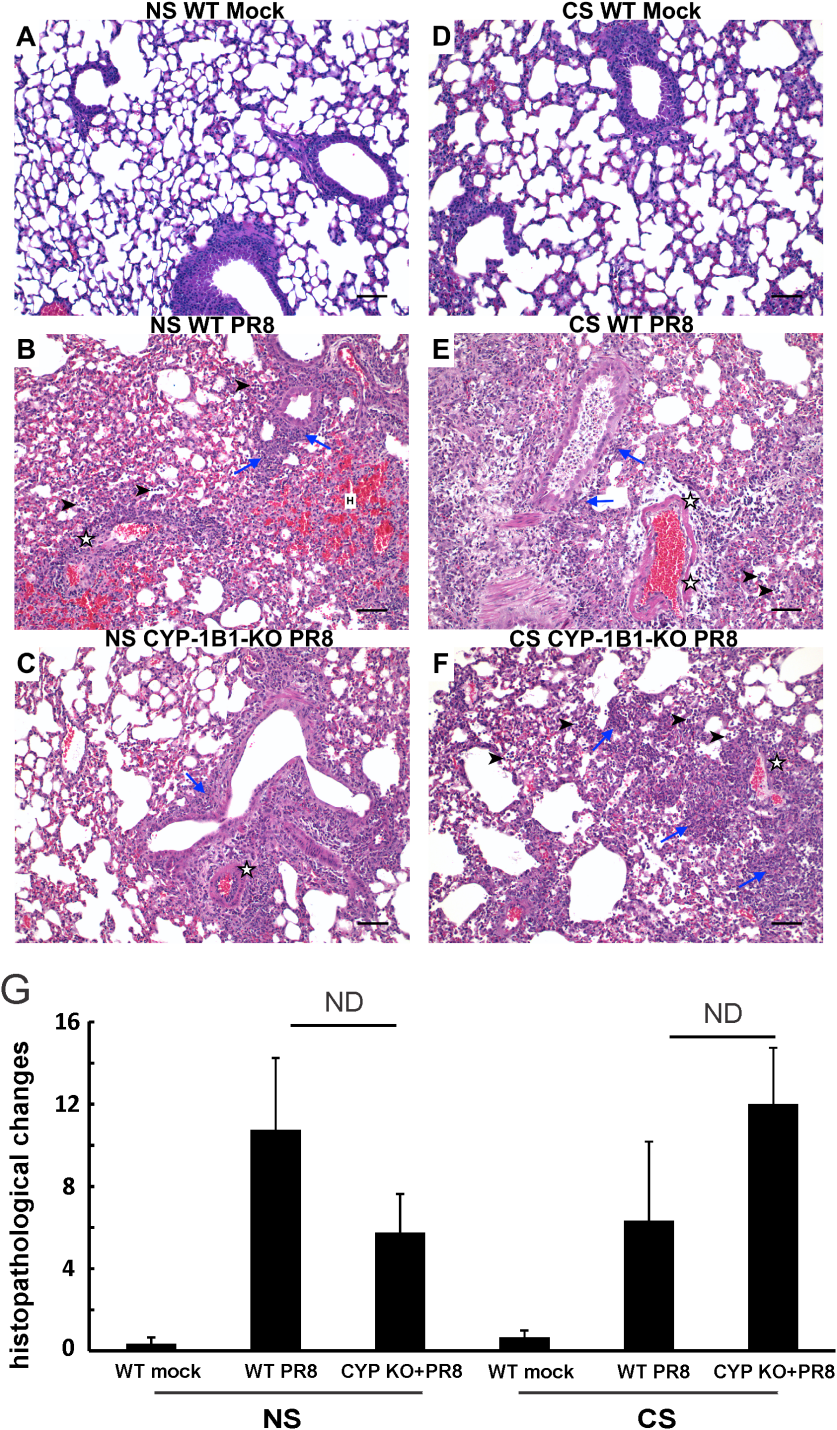
Effect of CYP KO on pulmonary histopathology during IAV infection in NS and CS-exposed mice. Each mouse was infected intranasally with 500 PFU of IAV. Mock-treated mice were inoculated with PBS. Animals were sacrificed 5 days after infection, and lung tissue was harvested. Lung tissue sections prepared from the infected mice were fixed, processed, and stained with hematoxylin–eosin **(A–F)**. Histopathologic evaluation and scoring of IAV infection were determined by a blinded pathologist **(G)**. Compared to the open alveolar spaces in healthy lungs of uninfected of mice with no CS (**A**, NS Mock), the lungs of mice with IAV infection **(B, C, E, F)** contained bronchiolar inflammatory infiltrates (blue arrows) that frequently spilled over into the adjacent alveolar spaces (arrowheads). The interstitial space surrounding small and large caliber vessels was also expanded by perivascular edema and lymphocytic infiltrates. Lesions in the lungs of IAV-infected mice featured distinct evidence of diffuse alveolar damage (DAD) characterized by denuded bronchial epithelium (asterisks), alveolar inflammatory infiltrates (arrowheads), and marked intra-alveolar fibrin (arrows). The lungs of healthy, uninfected mice subjected to CS (**D**, CS Mock) were also histologically normal, with open bronchi/alveoli and minimal alveolar edema. Scale bar = 50 µM (20X). The image shown is representative of 5 mouse lungs from each group; total mice= 30 in this experiment.

Lung tissues were evaluated for histopathological changes using H&E staining (**Figure 4**). At day 5 p.i., IAV-infected mice displayed a typical histopathological pattern for viral pneumonia, including diffuse alveolar damage (DAD) such as marked alveolar edema, fibrin exudation, hemorrhage, and prominent hyaline membrane formation. There was severe inflammation of bronchi and bronchioles characterized by epithelial cell necrosis and sloughing with partial to complete airway obstruction by intact and degenerate neutrophils and cellular debris, and varying degrees of acute intra-alveolar edema and/or hemorrhage. Lung histopathology of mock-infected control mice was unremarkable and within normal limits. Histopathologic scoring of these cardinal features of IAV infection was evaluated by a pathologist (CAM) blinded to the treatment groups. No significant difference was found in histopathologic scores between CYP KO+IAV PR8 and WT groups in both NS and CS mice. The results suggest that while CYP KO influenced immune cell influx and inflammation, as reflected in cell counts, BALF protein levels, LBR, and potentially histopathologic scores, the precise innate mechanism driving these changes remains uncertain.

### CYP1B1 KO induced early IFN-γ production in the lung during IAV infection

**Figure 5:**
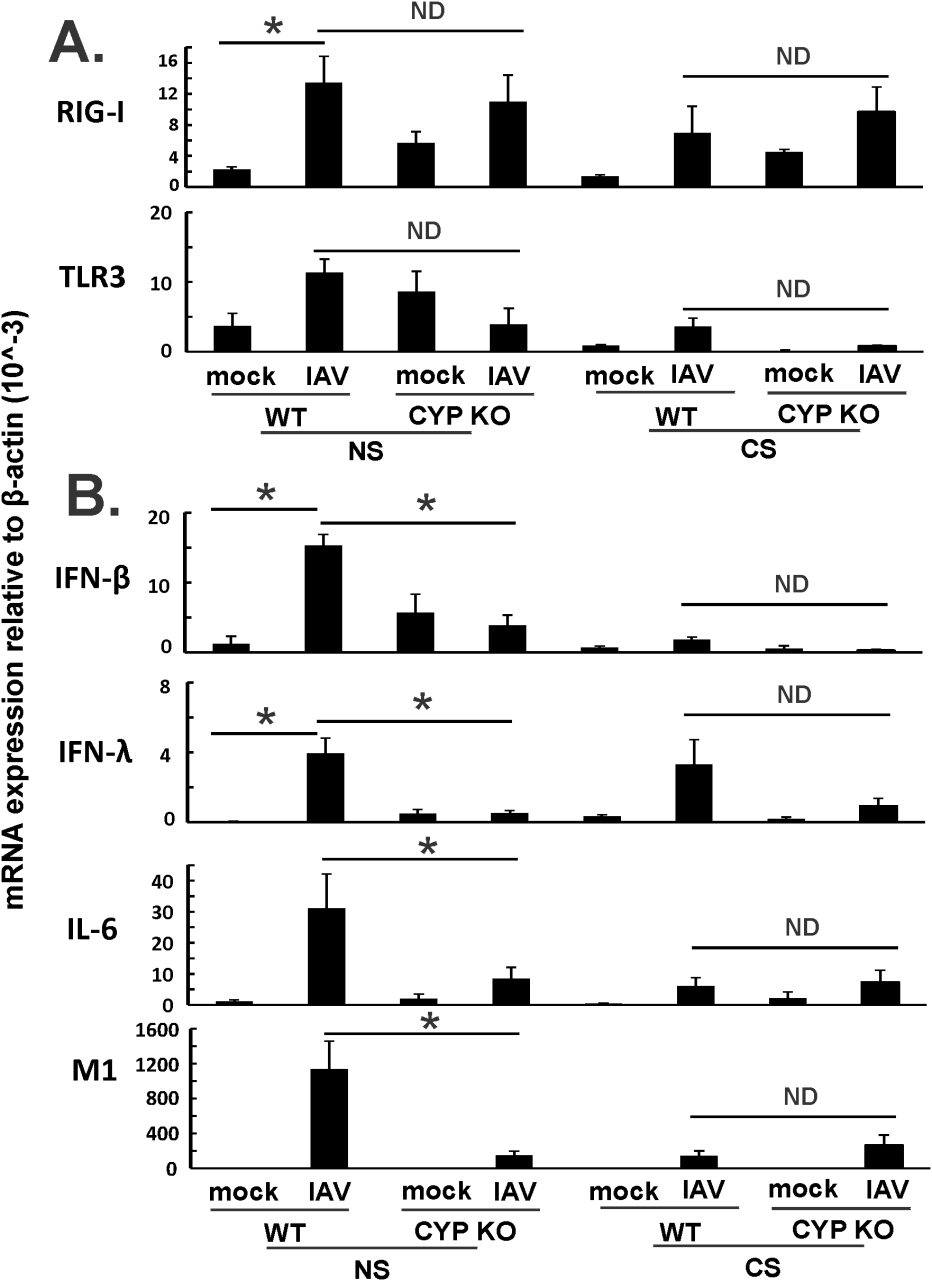
Innate immune responses to influenza infection in mouse lung. Each mouse was infected intranasally with 500 PFU of IAV. Mock-treated mice were inoculated with PBS. Mice were infected with 500 PFU of IAV. The mice were sacrificed at day 5 p.i. and **(A)** RIG-I, TLR3, and IAV M1 protein mRNA levels **(B)** cytokines IFN-β, IFN-λ and IL-6 mRNA levels in the lungs were assessed by qRT-PCR and normalized by β-actin levels. Data are expressed as mean ± SEM (*n* = 5; total mice = 40 in this experiment). * denotes significant difference between the two groups, *p* < 0.05. ND, no significant difference between the two groups. Statistical significance was determined by one-way ANOVA with Tukey test for multiple comparisons.

In order to examine additional mechanisms whereby CYP KO improved outcomes in IAV-infected mice, we measured expression of the pattern recognition receptors that recognize viruses and their downstream interferon expression during IAV infection in all mouse groups. Mice were inoculated intranasally with a single dose of the IAV PR8 strain (500 PFU). Lung tissues and BALF were collected at 5 days after infection. mRNA or protein expression was determined by qRT-PCR or multiplex immunoassay, respectively. CYP KO mice had similar RIG-I and TLR mRNA induction by virus compared to WT mice in both NS and CS mice (**Figure 5A**). For NS mice, CYP KO mice had significantly less IFN-β and IFN-λ than those in WT mice (**Figure 5B**), which might explain the decrease in lung injury seen in these NS CYP KO IAV mice (LBR, **Figure 3B**). As we have previously demonstrated (8, 34), CS-exposed WT mice had suppressed IFN-β and IL-6 mRNA induction by IAV compared to NS WT mice (**Figure 5B**; NS WT PR8 vs. CS WT PR8). NS WT PR8 mice had the most robust induction of the proinflammatory cytokine IL-6 mRNA induction among all the groups (**Figure 5B**). NS CYP KO IAV mice expressed significantly less IL-6 compared to the NS WT IAV group, and again, this may have contributed to the decrease in lung injury seen in NS CYP KO IAV mice (LBR, **Figure 3B**). In terms of viral RNA expression, the NS WT IAV mouse group had the most IAV M1 protein mRNA expression of any treatment group, while the NS CYP KO PR8 group had significantly less viral replication, which indicated CYP KO might be helpful in controlling viral replication in the lung, at least in NS mice (**Figure 5B**). CYP KO contained viral replication with reduced IFN-β, IFN-λ, and IL-6 mRNA levels in the NS mouse lung.

**Figure 6:**
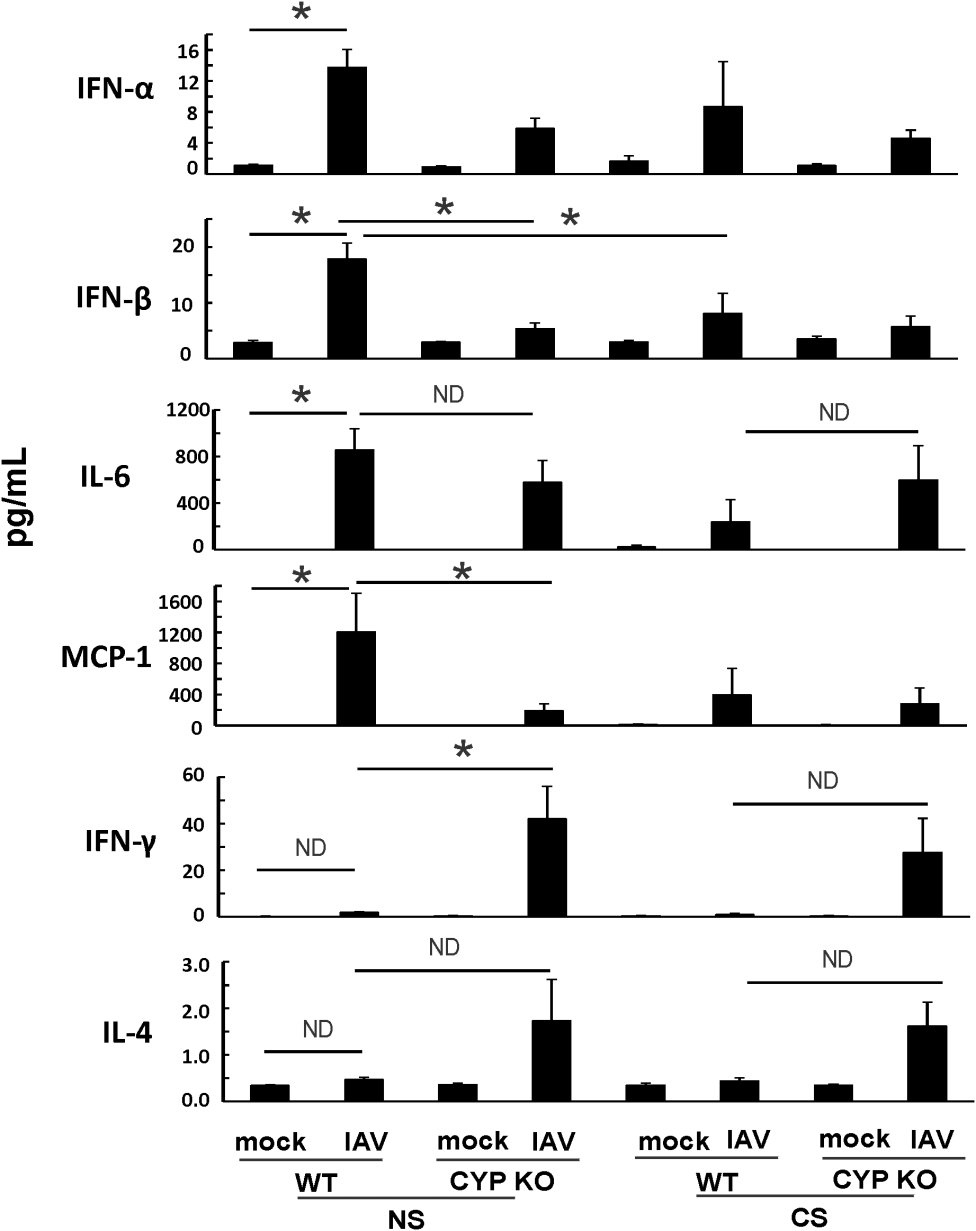
IAV-infected CS-exposed CYP KO mice had increased IFN-γ cytokine levels in BALF. Each mouse was infected intranasally with 500 PFU of IAV. Mock-treated mice were inoculated with PBS. BALF were harvested at day 5 post-infection. Mock treated mice were inoculated with PBS. Cytokine protein levels were determined by multiplex immunoassay. Data are expressed as mean ± SEM (*n* = 5 per group; total mice = 40 in this experiment). *denotes significant difference between the two groups, *p* < 0.05. ND, no significant difference between the two groups. Statistical significance was determined by one-way ANOVA with the Tukey test for multiple comparisons. NS WT PR8 vs NS CYP PR8 of MCP-1preotein was determined by unpaired student t test.

**Figure 7:**
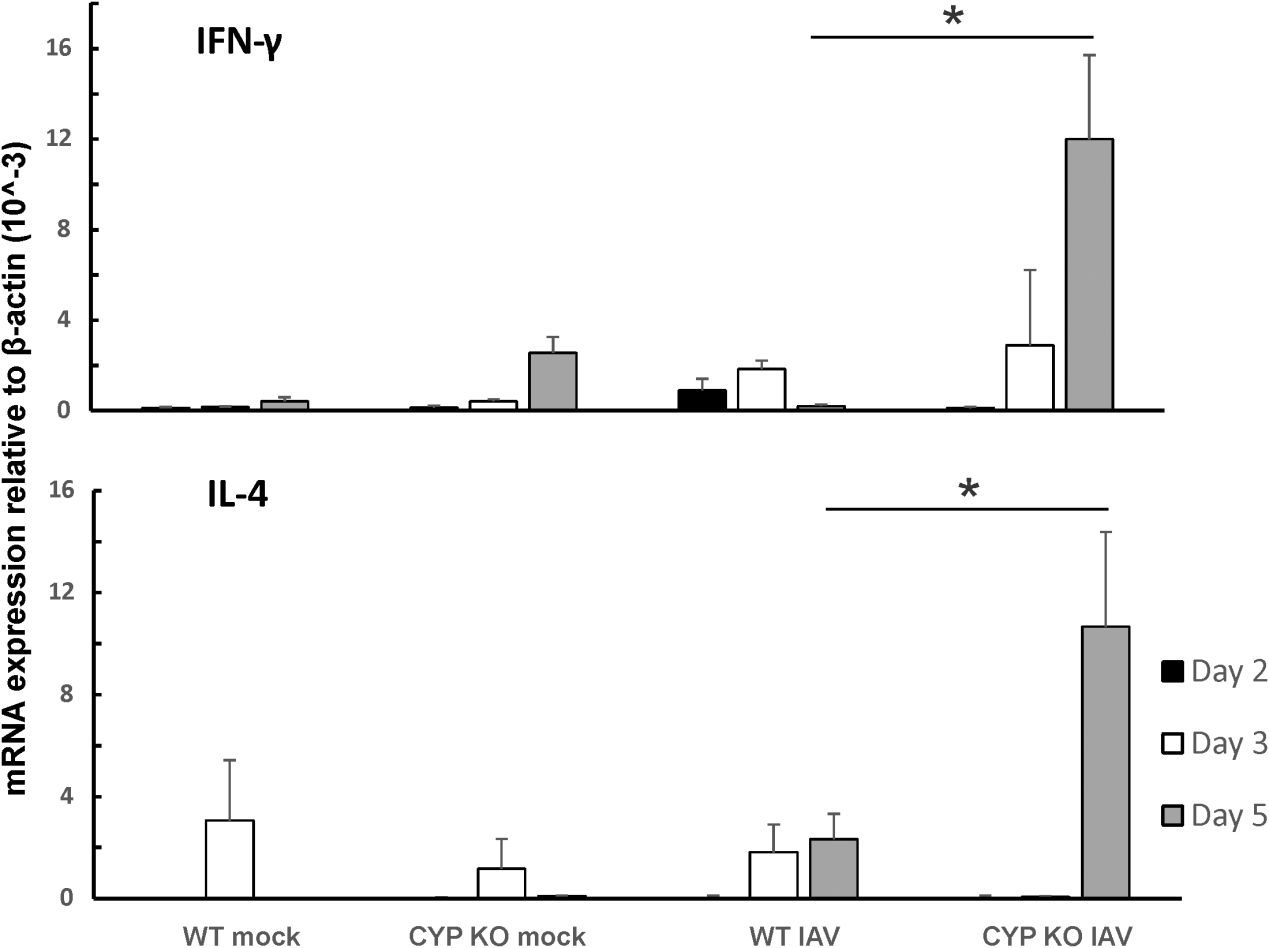
IFN-γ and IL-4 were dominantly induced by IAV infection in CYP KO NS mouse lung. Each mouse was infected intranasally with 500 PFU of IAV. Mock-treated mice were inoculated with PBS. The mice were sacrificed at days 2, 3, and 5 p.i., and IFN-γ and IL-4 mRNA levels in the lungs were assessed by qRT-PCR and normalized by β-actin levels. Data are expressed as mean ± SEM (*n* = 5; total mice = 60 in this experiment). * denotes significant difference between the two groups, *p* < 0.05. ND, no significant difference between the two groups. Statistical significance was determined by one-way ANOVA with Tukey test for multiple comparisons.

Since both IFN-β and IFN-λ mRNA expression levels were low, we next sought to find out the cause of the immune cell influx at day 5 p.i. as we showed in **Figure 3A**. We measured pro-inflammatory cytokine protein levels in mouse BALF in all groups using multiplex immunoassay (**Figure 6**). IFN-β protein induction by IAV was lower in CYP KO mice relative to WT cohorts in both NS mice (**Figure 6**). Cytokine IL-6 protein levels were similar among all infected groups. Cytokine MCP-1 protein level induction by IAV was also reduced in CYP KO mice. Remarkably, IFN-γ levels were significantly increased in CYP KO mice relative to those seen in WT mice. Interestingly, the anti-inflammatory cytokine IL-4 protein levels were also significantly induced in CYP KO regardless of NS or CS (**Figure 6**). Thus, these results demonstrate that CYP KO in mice boosted the pro-inflammatory cytokine IFN-γ response and anti-inflammatory cytokine IL-4 induction at day 5 after IAV infection. To determine the early kinetics of IFN-γ induction in CYP KO mice, we infected NS WT and KO mice and collected lungs at days 2, 3, and 5 after infection. We found that IFN-γ was similarly induced in IAV-infected WT and KO mice at day 3 p.i. (**Figure 7**). At day 5 p.i., CYP KO produced significantly greater IFN-γ mRNA in response to infection than IAV-infected WT mice did. IL-4 mRNA induction was also significantly enhanced by viral infection in KO mice at day 5.

### Lungs of NS CYP KO mice contained more myeloid cells and IFN-γ-producing NK cells after IAV infection

**Figure 8:**
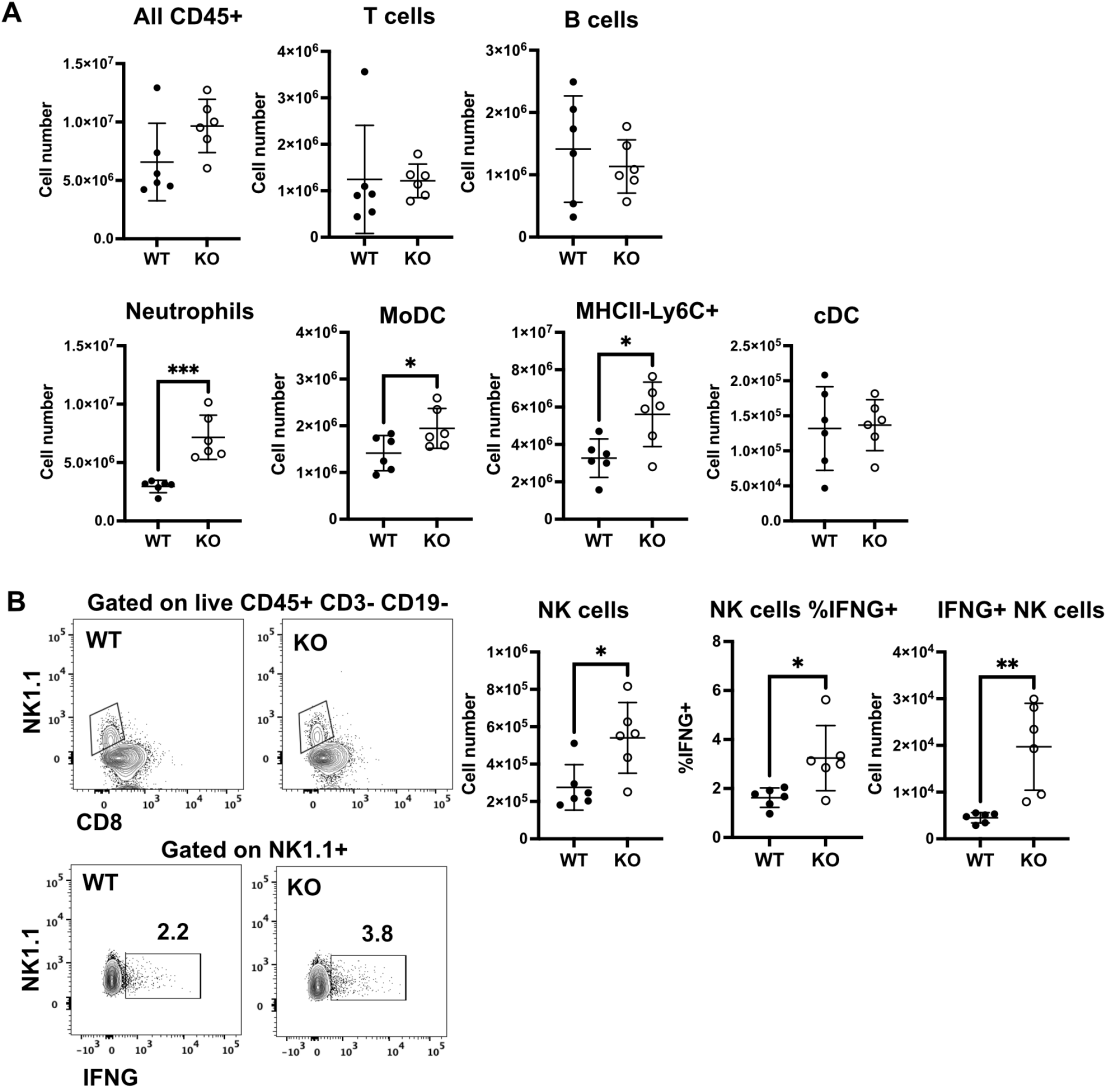
Increased numbers of IFN-γ ^+^ NK cells in lungs of NS KO mice. Each mouse was infected intranasally with 500 PFU of IAV. Lung cells were isolated at day 5 post-infection. **(A)** Numbers of the indicated lymphocytes and myeloid cell subsets in WT and KO mice. **(B)** Gating of NK cells as live CD45^+^CD3^–^CD19^–^NK1.1^+^ (upper panels) and intracellular IFN-γ in NK1.1^+^ cells (lower panels). Number of NK cells and frequency, and number of IFN-γ + NK cells. The graphs show mean ± standard deviation (*n* = 6; total mice = 12 in this experiment). Statistical analysis was performed by an unpaired two-tailed student t-test for each cell type. Significant differences between the two groups were denoted as *p < 0.05. **p < 0.01. ***p < 0.001.

To determine which cell(s) contribute to the increased IFN-γ, total lung cells were isolated at 5 days after IAV infection of NS mice. We measured the numbers of lymphocytes and myeloid cells and the presence of intracellular IFN-γ in the lungs of WT and KO mice by flow cytometry. Total CD45^+^ leukocyte numbers in the lung trended higher in KO mice, as we found in BALF in **Figure 3A** (**Figure 8A**). While numbers of B and T lymphocytes did not differ between WT and KO mice, CYO KO mice also had significantly higher numbers of myeloid cells, including neutrophils, MHCII^+^ monocyte-derived DCs (MoDC, MHCII^+^CD11c^+^ Ly6C^+^ CD64^+^), and MHCII^–^ monocyte-macrophages (MHCII^–^CD11c^+^Ly6C^+^CD64^+^) during IAV infection (**Figure 8A**). However, neutrophils and moDC are not major IFN-γ producers. Lung NK cells are a major source of IFN-γ in early stages of IAV infection (44). The number of NK cells and the frequency and number of IFN-γ+ NK cells were significantly higher in KO mice than in WT cohorts (**Figure 8B**). These data suggest that innate IFN-γ-producing NK cells are involved in the enhanced production of IFN-γ in CYP KO mice after IAV infection.

### IFN-γ is required but not sufficient for protection of CS-exposed mice against lethal influenza virus infection

**Figure 9:**
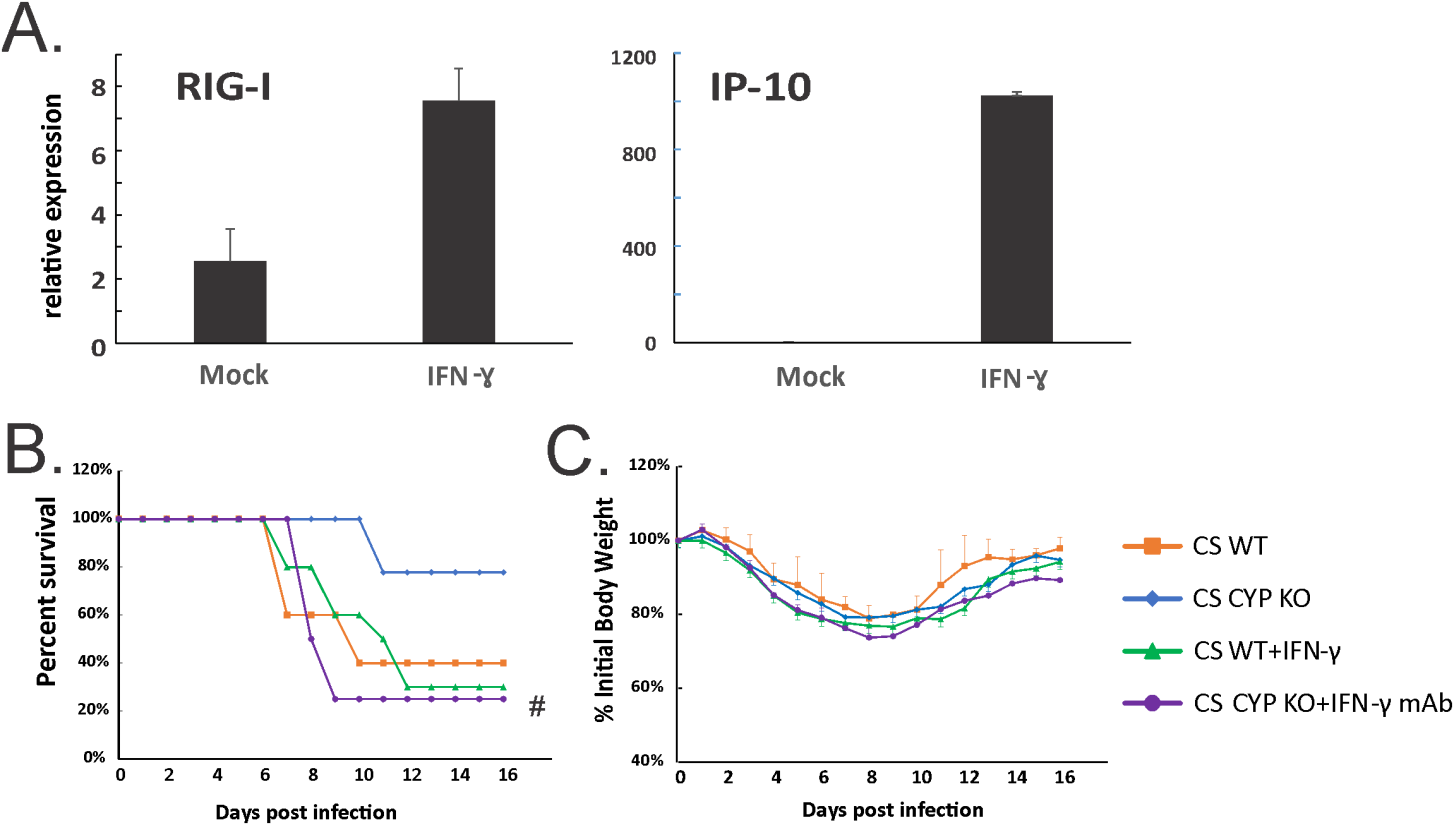
Neutralization of IFN-γ abolished enhanced survival in CS-exposed CYP KO mice during lethal IAV infection. **(A)** IFN-γ administration induced robust RIG-I and IP-10 mRNA induction in mouse lung. C57BL/6 mice were administrated with IFN-γ (5 µg/mouse) intratracheally in a total volume of 50 μl in PBS. After 6h, the mouse lungs were collected. mRNA levels were assessed by qRT-PCR and normalized to β-actin. Bar graph represents mean ± standard deviation (*n* = 3; total mice = 6 in this experiment). Mortality **(B)** and body weight **(C)** during lethal IAV infection in CS mice. The mice were intranasally inoculated with IAV at 500 PFU/mouse. CS WT+IFN-γ group received IFN-γ (5µg/mouse) intratracheally at day 3 p.i. CS CYP KO+ IFN-γ mAb received anti-IFN-γ mAb (clone XMG1.2; BioXcell, Lebanon, NH) intraperitoneally (600 µg/mouse) on days 2, 4, and 6 p.i. Mortality and body weight were monitored daily. Body weight data were normalized to each mouse’s starting body weight. Data are expressed as mean ± standard deviation (*n* ≥ 7 for each group; total mice = 36 in this experiment). #denotes significant survival rate difference between the CS CYP KO and CS CYP KO+ IFN-γ mAb, *p* < 0.05. Survival rate significance was determined by the log-rank test.

To determine whether IFN-γ induction may play an important role in the protection of the CS-exposed animals from IAV infection in KO mice, we next examined whether exogenous IFN-γ administration improved outcomes in CS-exposed IAV-infected WT mice. First, we confirmed that recombinant mouse IFN-γ (BioLegend, San Diego, CA) administration induced innate immune responses in non-infected animals. Mice were lightly anesthetized with isoflurane and treated with IFN-γ (5 µg/mouse) intratracheally in a total volume of 50 μl in PBS. Mock groups were sham-treated with a single dose of intranasal sterile PBS solution (diluent). We found that IFN-γ induced robust innate immune responses in the mouse lung, including increased mRNA of RIG-I. Significantly, mRNA levels of interferon gamma-induced protein 10 (IP-10, a cytokine downstream of IFN-γ) were induced 426-fold over mock by IFN-γ administration (**Figure 9A**). We next evaluated the effect of IFN-γ administration on mortality and weight loss in CS-exposed, IAV-infected WT mice. We experimentally determined an LD50 for CS-exposed mice and discovered this dose was 500 PFU/animal. We used this dose in CS-exposed mice with or without IFN-γ administration (**Figures 9B, C**). IFN-γ was given to mice intratracheally at day 3 after IAV infection. We found IFN-γ-treated CS WT mice had almost the same mortality as untreated CS WT IAV-infected mice (**Figure 9B**), which suggested IFN-γ administration alone did not protect the CS-exposed WT mice from lethal IAV infection. We also tested whether blocking IFN-γ abolishes the protection in CYP KO mice during IAV infection. Mice were intraperitoneally injected with 600 µg of anti-IFN-γ monoclonal antibody (mAb) on days 2, 4, and 6 p.i. IFN-γ neutralization significantly decreased survival at the LD_50_ for CS-exposed mice (80% for CS CYP KO vs. 20% for CS CYP KO+IFN-γ Ab mice). The data suggested that IFN-γ was required but not sufficient for protection of CYP KO CS mice during lethal IAV infection.

## Discussion

In this report, we have demonstrated that CYP1B1 is one of the most highly upregulated (sixfold over nonsmokers) genes in lung epithelial cells obtained from smokers (**Table 1**). With regard to IAV infection, we found that CYP1B1 mRNA expression is induced during IAV infection, and further enhanced by CS exposure. These results suggest a correlation between increased CYP1B1 expression and increased mortality during IAV infection with CS exposure.

We discovered that CYP KO significantly increased survival during IAV infection in CS-exposed mice. Further investigation revealed that CYP KO significantly increased IAV-induced total immune cell numbers in BALF without causing additional lung injury. Notably, we found that CYP KO significantly increased early IFN-γ induction in the lungs at day 5 p.i. in NS mice. IFN-γ, also known as type II IFN, is a potent antiviral cytokine that plays multiple roles in both innate and adaptive immune responses to IAV infection. Mice lacking IFN-γ or its receptor are highly susceptible to infectious diseases, emphasizing its importance (45). IFN-γ stimulates the recruitment of both innate and adaptive leukocytes to the infection site (46). The CYP1B1 KO mice’s increased IFN-γ induction could be the cause of their higher BALF total immune cell counts.

Normal inflammation is a protective response by immune cells to fight against pathogens. The purpose of the response is to eliminate the pathogen while limiting the damage to the host. In mild infection, the host has a limited or moderate response with little tissue damage, and thus, the disrupted homeostasis is restored rapidly. Either uncontrolled (usually in pandemic IAV infection) or suppressed (in immunosuppressed patients) inflammation can be dangerous to the host. There are several instances where impairment of immune cell recruitment results in worse outcomes of IAV infection. Alveolar macrophage (AM) depletion is associated with enhanced lethality of IAV infections in mice (47). Neutrophil depletion impairs the response to IAV infections, leading to increased viral load and impaired T-cell responses (48). As we have shown previously, there is suppressed immune cell recruitment in the lungs of CS exposed, compared to NS mice during IAV infection (8, 34). Thus, CS impairs cellular antiviral activity at an early stage of IAV infection (17). In our current work, we propose that the enhanced recruitment of immune cells to the lung of CYP KO mice may partially reverse the immune defect in CS-exposed mice but may not be beneficial in NS mice where recruitment is normal. This may partially explain why we found significant protection of CYP KO in CS mice but not in NS mice. CYP KO in CS mice restored the CS-suppressed innate responses but did not promote excessive inflammation and ALI in the lungs. For NS mice, CYP KO did increase the immune cell infiltration in the lung (**Figure 3A**), but lung damage and pathological score in the lung were both decreased in CYP KO mice as we show in **Figures 3B** and **4G**. This controlled enhancement of immune cell infiltration likely contributed to more effective pathogen clearance as demonstrated (**Figure 5B**), which in turn led to a slight reduction in mortality during the later stages of the disease (**Figure 2B**). Thus, though there was enhanced immune cell migration, the resultant increased viral clearance resulted in no enhancement of lung damage. Thus, CYP KO in NS mice corrected the immune response to a more optimal level, as it did not increase deleterious inflammation and mortality. In contrast, IFN-β administration to NS mice caused more lung damage during IAV infection, as we have shown in our prior publication (8). These results further supported the concept that normal and appropriate inflammation may be necessary and beneficial for the survival of the mice during seasonal IAV infection.

For WT NS mice, innate lymphoid cells, particularly NK cells, were the dominant producers of IFN-γ, both in terms of numbers of cells and amount of cytokine produced, at the early stage after IAV infection (44, 49, 50). In IFN-γ KO mice, DCs and T cells show decreased migration to the lymph nodes and limited influenza-specific responses in the lung. This is rescued by the adoptive transfer of WT NK cells; NK cell-derived IFN-γ alone is sufficient for T cell activation during influenza infection, while T cell-derived IFN-γ is complementary (51). Our data showed here that NK cells are likely a major producer of early IFN-γ release in CYP KO mice (**Figure 8**).

We confirmed the importance of IFN-γ for CYP KO enhanced survival by adding IFN-γ antibody to CYP KO mice. IFN-γ antibody treatment of CYP1B1 KO mice completely abolished the higher survival rate in the CS-exposed IAV-infected mice. Additionally, IFN-γ administration to CS-exposed WT mice did not increase survival rates as seen in CYP KO mice. The data suggested that early IFN-γ is one of the multiple factors involved in improved outcomes in KO mice. Thus, for CS-exposed CYP1B1 KO mice, IFN-γ is necessary but insufficient for enhanced protection against fatal IAV infection. Previously, one group found that CSE inhibits the IFN-γ–induced antiviral cell signaling, supporting the concept that exposure of the human airway to CS directly impairs antiviral defenses (52). The inhibition results in increased incidence, duration, and/or severity of IAV infection. Augmented IFN-γ induction in KO mice might reverse the CS inhibitory effects, especially during the early stage of infection.

In IAV-infected mice, there is a notable increase in the accumulation of NK cells in the lungs. This is mainly due to increased NK cell recruitment with a small contribution of enhanced local proliferation of pulmonary NK cells (53). Airway epithelial cells are the lining cells of the airways and produce various chemokines in response to stimuli like viral infections or allergens, which then attract immune cells like NK cells (54). Chemokines commonly associated with NK cell recruitment include CXCL10, CXCL9, and CCL5 (RANTES) (55). We found that, at day 5 p.i., CYP KO produced significantly greater IFN-γ mRNA and protein in response to infection than did IAV-infected WT mice. The number of NK cells and the frequency and number of IFN-γ+ NK cells were significantly higher in KO mice than in WT cohorts (**Figure 8B**). It is possible that increased NK recruitment to the lung and elevated IFN-γ production in NK cells are potential mechanisms for improving CYP KO mouse survival in IAV-infected, CS-exposed mice.

Aryl hydrocarbon receptor (AhR) is a transcription factor that plays a crucial role in regulating gene expression in response to both environmental stimuli and endogenous ligands. Upon activation, AhR translocates into the nucleus, where it modulates the expression of numerous target genes. These genes include the AhR repressor, detoxifying enzymes such as CYP1A1 and CYP1B1, and various cytokines (56). During IAV infection, AhR activation enhances neutrophil recruitment to the lungs and upregulates the expression of IFN-γ and inducible nitric oxide synthase (iNOS), contributing to the host immune response (57, 58). In some cases, CYP1B1 inhibitors appear to increase AhR activity, particularly in the context of certain metabolic pathways or under specific conditions. This is because CYP1B1 is involved in the metabolism of various compounds, including those that activate the AhR, and inhibiting CYP1B1 alters the availability of these ligands, leading to changes in AhR activity (59, 60). We propose that CYP KO, like CYP1B1 inhibitors, activates AhR, enhances NK cell, neutrophil, and MoDC recruitment to the lungs (**Figure 8**), and upregulates immune cytokine expression, including IFN-γ (**Figures 6**, **7**).

Taken together, our work and the current literature demonstrate that CYP1B1 is one of the most highly upregulated genes in human and mouse lungs exposed to CS. CYP1B1 KO in mice is protective for CS-enhanced susceptibility of smokers during influenza infection. IFN-γ is important in the mechanism of protection. IFN-γ is required but is not sufficient for the protection of CS-exposed CYP KO mice against lethal influenza virus infection. Our study provides new insight into how CS worsens outcomes in IAV infection through inducing CYP1B1 and dysregulating the immune response. The results will not only advance our understanding of the pulmonary host response against viral pathogens but may also be directly applicable to other commonly encountered inflammatory conditions within the lung, including tuberculosis, asthma, and COPD. Our detailed mechanistic studies will also identify new opportunities for intervention that could improve outcomes in IAV infection, particularly in smokers.

## Data Availability

HBEC RNA sequencing data have been deposited in the National Center for Biotechnology Information (NCBI) Gene Expression Omnibus and are accessible through Gene Expression Omnibus Series accession number GSE120908.
